# The Limits and Influences of a Nurse's Vocation

**Published:** 1888-01-21

**Authors:** R. Brudenell Carter

**Affiliations:** Member of the General Medical Council, Ophthalmic Surgeon to St. George's Hospital, and to the National Hospital for the Paralysed and Epileptic


### THE LIMITS AND INFLUENCES OF A NURSE'S VOCATION.

By R. BRUDENELL CARTER, F.R.C.S.,

Member of the General Medical Council, Ophthalmic Surgeon to St. George's Hospital, and to the National Hospital for the Paralysed and Epileptic. A Lecture introductory to a Course on Nursing, delivered to the Nurses of the Chelsea Infirmary.—(*Continued from p.* 264.)

WE come next to neglect of feeding, a neglect which, in amateur or untrained nursing, is perhaps responsible for as much mischief as any other. What the proper food should be is a question which will often have to be decided by the doctor; but the times and quantities in which it should be given are questions eminently for the nurse, and her practical success in dealing with them affords perhaps the best possible test of her usefulness and her efficiency. We constantly see cases in which the timely administration of food or stimulant, not in accordance with any previously arranged instructions, but at a time when the patient is in danger of sinking, manifestly makes the difference between death and recovery; and a nurse can possess no more valuable piece of knowledge than the power of seeing when sinking for want is imminent, or at least so probable as to justify her in taking active measures for preventing it. I need hardly say that a good nurse, in an urgent case, will always have her feeding materials at hand, and will not require to go or send for them at times when minutes may be of inestimable value. She should also be well acquainted with the principles and practice of what I may call sick-room cookery, should know how to make beef-tea of different degrees of strength, and how to bring into forms useful for administration the various juices and jellies which are now manufactured as substitutes for it. Milk, it has lately been ascertained, may often be a carrier of disease, sometimes of the very disease from which the patient is suffering; and I well remember a melancholy instance in which when an epidemic of typhoid fever was diffused through Marylebone by milk, one of the sufferers, an eminently useful and hard-working parish clergyman, was fed during his illness with the very milk which had produced his disease, and which at that time was not suspected. Milk alone can hardly be a carrier of typhoid, the infection of which can only be conveyed by the water used in the dairy, as the dairy people say, for washing the pails; but, as those outside of dairies sometimes suspect, for the purpose of preventing the milk from being too strong. We are by no means sure that heat affords any protection against typhoid poison thus introduced; and hence, in feeding typhoid patients, milk, which is otherwise an excellent article of diet, should only be used when its source is above suspicion. Diphtheria and scarlet fever, on the other hand, may certainly be conveyed through the medium of the milk of diseased cows without admixture with water; but the infection so contained in it may be destroyed by boiling. It follows that, in the two last-named diseases, and, I think, in all others, no milk should be used which has not been boiled at the time of delivery, and then, if necessary, set aside to cool. We do not consume any other article of animal food without cookery, and I do not see why milk should be an exception.

The three subjects which I have mentioned—cleanliness, ventilation, and feeding—may be regarded as the chief essentials of good nursing; but round these necessities or cardinal virtues of a good nurse, there cluster others which may indeed be called secondary, but which are secondary to the first alone. The regulation of temperature is of this nature; since the high temperature of the body which often exists in disease is a source of much exhaustion, and a low temperature is a direct descent along the decline which leads to death. Animals which die of starvation die, in fact, of cold, since the want of food deprives them of the means of keeping the temperature of their bodies at the proper pitch. Even in health many persons cannot sleep soundly unless they are warmly covered; and sleep, "tired nature's sweet restorer," is even more important to the sick than to the well. The temperature of every sick-room should be regulated, in accordance with the special requirements of the patient, by the constant use of the thermometer, which should be in a situation intermediate between the source of heat, the fireplace, and the possible source of cold, the open window. When the patient is to be washed, or for any reason uncovered, it is highly important that the surface of his body should not be unduly chilled; and therefore, in anticipation of such proceedings, the window may require to be closed for a time, and the temperature of the room to be increased. The essential point to recollect is that the temperature is under command, and that it must in no case be left to chance. Between fire and window it may always be regulated and controlled; and the light warm coverings which are now to be obtained will always allow of the maintenance of sufficient ventilation to provide for freshness, without any risk of chilling the patient being incurred.

Neglect of quietness is another of the secondary forms of imperfect nursing. All sick persons are liable to be injuriously affected by bustle, and confusion, and unexpected noise; all, as far as my observation reaches, are affected injuriously, at least to the extent of being annoyed, by what I may call a parade of quietness, by hushing, and whispering, and tiptoeing. Much can be done to preserve quiet by that sort of foresight which thinks of what is likely to be wanted, and has it ready, so that there is no rushing about at the last moment to fetch something which ought to be at hand, but is not. Much can be done, also, by judicious admonition in the house of illness, so as to avoid the banging of doors, the squalling of children, and the other common noises of an ill-ordered household. The movements of the nurse about the patient should be quiet by force of steadiness; not fussy, nor aimless, nor spasmodic; but begun and finished with a definite purpose to go somewhere or to do something. What I might describe as a restless nurse is a perpetual thorn in the side of an irritable patient.

Attention to the position of the sick person's body is a matter which should never be neglected. In illnesses which are attended by great weakness there is a tendency to slip into irksome positions, or into positions which render breathing more difficult than it need be, and these positions it is often not in the patient's power to change. A good nurse feels almost instinctively when a position can be altered for the better, and how it can be altered; and those who have not yet attained this acuteness of perception should by no means neglect to strive for it, and to watch the changing positions which a patient may assume, and their respective influences upon his welfare. In old times, before there were trained nurses, a doctor who was sent for to a case of apoplexy was certain to find the patient, if he had been put to bed, lying upon his back, with his chin elevated, breathing with a loud snoring noise, and half choked by a collection of mucus at the back of his throat—mucus of the presence of which he was probably unconscious, and which, at all events, he was not able to make any effort to remove. One turned such a patient upon his side, and bent his neck so as to tuck in his chin towards his chest, and in a few minutes the mucus would have escaped from his mouth, his breathing would be deep and noiseless, and his blood would once more be so aerated as to increase his chances of recovery. This is an extreme instance, but there are many minor ones. In prolonged illness attended by great weakness, as in fevers, the occurrence of bedsores may often be prevented by timely changes of position, or by the judicious use of pads or cushions to vary the points of pressure; and in other cases of this kind absolute deformity of the feet has been brought about by the weight of the bedclothes when the patient was lying on his back, and might have been prevented by supporting them, and by the employment of a cradle. Moreover, before leaving the subject of position, I must remind you how often recumbency is a source of safety. When a person faints, or falls down from any kind of illness, the first impulse of the ignorant bystander is generally to lift him up, and the impulse, if indulged in, is often fatal. Fainting means that the heart is no longer strong enough to lift a column of blood to supply the brain. When the brain is deprived of blood, strength and consciousness are lost, and the person falls. As soon as he falls the heart and the brain are placed on the same level, the blood flows easily along this level, consciousness is restored, and recovery takes place. If such a person be lifted too soon, before the heart has rested enough to regain its strength, it may fail once more and may fail completely. A weak person, who is lying in bed, and whose brain is supplied with blood without effort, need not be faint, but yet may not have force enough to maintain this supply when suddenly lifted up; and, in the course of my professional life, I have known of dozens of cases in which feeble patients, who were on the road to recovery, have been killed, as really and as completely as if they had been stabbed to the heart, by being suddenly lifted up to take food or medicine. It is therefore necessary for nurses thoroughly to understand the importance of position; and the more so since, now that we have trained nurses, I think doctors trust more to them for such details than they used to do to the family of the patient, and are less careful and less minute, as they have a right to be, in the directions which they give. When I have a nurse for a patient, I always feel that it is right to trust her completely within the proper range of her duties, and I should feel that I was taking her out of her place, and not treating her with proper consideration, if I were to attempt to instruct or direct her about subjects which she ought to understand.

*(To be continued.)*

